# Molecular Regulation of FOXO1 and Its Pathophysiological Significance in Endometriosis: A Narrative Review

**DOI:** 10.3390/antiox15010003

**Published:** 2025-12-19

**Authors:** Hiroshi Kobayashi, Hiroshi Shigetomi, Miki Nishio, Mai Umetani, Shogo Imanaka, Hiratsugu Hashimoto

**Affiliations:** 1 Department of Gynecology and Reproductive Medicine, Ms.Clinic MayOne, 871-1 Shijo-cho, Kashihara 634-0813, Japan; mikin.yaruki10000.boshi@gmail.com (M.N.); mai_umetani@yahoo.co.jp (M.U.); shogo_0723@naramed-u.ac.jp (S.I.); hiratsugu_hashimoto@yahoo.co.jp (H.H.); 2 Department of Obstetrics and Gynecology, Nara Medical University, 840 Shijo-cho, Kashihara 634-8522, Japan; 3 Department of Gynecology and Reproductive Medicine, Aska Ladies Clinic, 3-3-17 Kitatomigaoka-cho, Nara 634-0001, Japan

**Keywords:** apoptosis, decidualization, endometriosis, FOXO (Forkhead box O), senescence

## Abstract

Background: Endometriosis is a chronic inflammatory disorder that affects approximately 10% of women of reproductive age and exhibits tumor-like characteristics such as invasion, recurrence, and hormone-dependent proliferation despite its benign nature. Its pathogenesis is thought to involve hormonal imbalance, oxidative stress, hypoxia, immune dysregulation, and epigenetic alterations. This review summarizes how these factors contribute to lesion formation through intracellular signaling pathways, with a particular focus on the role of the stress-responsive transcription factor Forkhead box O (FOXO1). Methods: A comprehensive literature search was conducted using PubMed and Google Scholar without temporal restriction. Results: FOXO1 is a transcription factor that integratively regulates decidualization, cellular senescence, autophagy, and apoptosis. In the normal endometrium, under mild stress or hormonal stimulation, FOXO1 induces decidualization-associated genes (PRL, IGFBP1) and antioxidant enzymes, thereby promoting differentiation and survival. In contrast, in endometriosis, activation of the PI3K/AKT signaling pathway and an estrogen-dominant environment suppress the nuclear activity of FOXO1, leading to apoptosis resistance, accumulation of senescent cells, and chronic inflammation through the senescence-associated secretory phenotype (SASP). Moreover, depending on the intensity and duration of oxidative, metabolic, and environmental stress, FOXO1 drives distinct cellular fates—including decidualization, senescence, and apoptosis—thus contributing to the persistence and progression of endometriotic lesions. Conclusion: Dysregulation of the FOXO1-dependent cellular fate–control network plays a central role in the development of endometriosis. Elucidating the molecular mechanisms governing FOXO1 activity and its nuclear dynamics will be crucial for a comprehensive understanding of disease progression and for the development of novel therapeutic strategies.

## 1. Introduction

Endometriosis is a chronic inflammatory disease characterized by the ectopic presence of endometrial tissue outside the uterine cavity and is commonly associated with dysmenorrhea, chronic pelvic pain, and infertility [1,2]. It affects approximately 10% of women of reproductive age and significantly impairs quality of life, making it a major medical and social concern [1]. Although histologically classified as a benign disorder, endometriotic lesions often exhibit malignant-like features, including estrogen-dependent proliferation, local invasion, and frequent recurrence [3]. Owing to these characteristics, endometriosis has attracted increasing attention not only as a benign gynecological disease but also from a tumor-biological perspective [3].

The progression of endometriosis cannot be fully explained by retrograde menstruation of endometrial cells alone; rather, it is believed to involve alterations in the peritoneal microenvironment, immune dysregulation, and the intricate modulation of multiple intracellular signaling pathways [2]. Hormonal abnormalities such as estrogen dominance and progesterone resistance are characteristic of the disease and directly contribute to impaired decidualization, as well as to the proliferation and survival of endometriotic cells [2]. Chronic inflammation and oxidative stress stimulate the production of cytokines and inflammatory mediators, creating a vicious cycle in which inflammation and estrogen biosynthesis mutually reinforce each other [4,5]. In addition, persistent exposure to stress stimuli induces Deoxyribonucleic acid (DNA) damage, cell cycle arrest, senescence, autophagy dysfunction, and anti-apoptotic responses; inflammatory factors secreted by senescent cells suppress immune clearance, thereby facilitating the persistence of ectopic endometrial tissue [6,7]. Hypoxic conditions further promote angiogenesis and fibrosis, contributing to lesion stabilization and progression [8]. Moreover, aberrant epigenetic regulation has also been reported, highlighting the multifactorial nature of the disease [9,10,11].

Thus, endometriotic cells are exposed to diverse environmental stressors—including hormonal imbalance, oxidative stress, inflammation, hypoxia, and metabolic stress—and survive and proliferate by dynamically adjusting multiple signaling pathways in response [7]. However, the mechanisms by which these pathways interact to promote immune evasion, metabolic reprogramming, cell proliferation, senescence, apoptosis resistance, and fibrosis remain incompletely understood. A major limitation of current research is that most studies have focused on individual molecules or signaling cascades, with relatively few attempts to integrate these findings into a comprehensive framework of pathophysiology. Furthermore, emerging concepts such as cellular senescence have not yet been fully explored in this context [10]. There is also a lack of studies directly linking clinical phenotypes to molecular mechanisms, which poses challenges to translating these findings into practical therapeutic targets.

This review aims to provide an integrative overview of the major signaling pathways involved in the pathogenesis of endometriosis, with particular emphasis on their interplay in regulating cell proliferation and differentiation. By focusing on the FOXO (Forkhead box O) family of transcription factors as central hubs of stress response signaling, we seek to highlight their potential role in disease progression and propose future directions for research in this field.

## 2. Materials and Methods

### Search Strategy and Selection Criteria

Because this review encompasses diverse types of studies—including in vitro experiments, animal models, and clinical investigations—some degree of heterogeneity in content and quality was anticipated. Therefore, a narrative review format was adopted rather than a systematic review. Clear inclusion and exclusion criteria were established, and only studies deemed highly relevant to the topic were selected. The literature search was conducted using the keywords “endometriosis,” “decidualization,” “autophagy,” “cellular senescence,” “apoptosis,” and “FOXO,” combined with Boolean operators. Searches were performed in PubMed (https://pubmed.ncbi.nlm.nih.gov/) and Google Scholar (https://scholar.google.com/). The search period covered all available years up to July 2025, and only articles published in English, including original research and review papers, were considered. Additional relevant studies were identified through the reference lists of the retrieved articles. Although the primary focus was on studies involving the endometrium and endometriosis, research using other cell types or animal models was also included where appropriate, with such cases explicitly noted in the text. Publications not written in English were excluded.

## 3. Results

The FOXO family of transcription factors (FOXO1, FOXO3, FOXO4, and FOXO6) has been extensively studied as key regulators of diverse cellular fates, including cell-cycle control, stress responses, apoptosis, and cellular senescence. In particular, FOXO factors respond to various stresses, such as oxidative stress, nutrient deprivation, DNA damage, hypoxia, and inflammatory stimuli. Dysregulation of FOXO1 signaling has been reported in a wide range of pathological conditions, including cancer, metabolic disorders, and age-related diseases, underscoring its importance in disease development [12,13]. In Section 3.1 of this review, following an overview of FOXO transcription factors, we summarize the functions of FOXO—particularly FOXO1—and the mechanisms regulating their activity in cells and tissues of the normal endometrium as well as in endometriosis. In Section 3.2, we first outline the established signaling pathways involved in the regulation of FOXO family transcription factors and then describe the characteristic features of FOXO-related signaling in the endometrium and endometriosis. Finally, in Section 3.3, we integrate and discuss how FOXO transcription factors are differentially regulated across distinct pathological contexts of endometriosis.

### 3.1. Roles of FOXO in Endometrial Function and Its Involvement in Endometriosis

#### 3.1.1. Functions of FOXO and Its Role in the Endometrium

**Figure 1:**
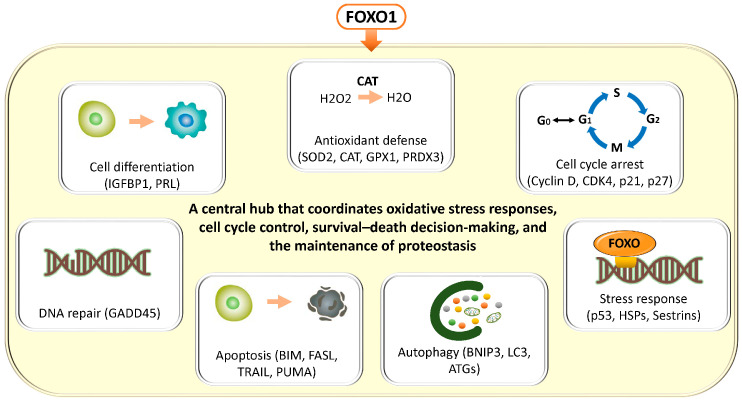
Diverse cellular stress responses and homeostatic regulatory programs mediated by FOXO transcription factors. FOXO transcription factors have been shown, through studies using a variety of cellular models, to integratively regulate multiple cellular programs involved in stress resistance and the maintenance of cellular homeostasis. FOXO promotes antioxidant defense through the regulation of genes such as SOD2, CAT, GPX1, and PRDX3, while inducing cell cycle arrest by modulating Cyclin D, CDK4, CDKN1A (p21), and CDKN1B (p27). In addition, FOXO supports DNA repair via GADD45 and regulates cell death signaling through the transcriptional activation of apoptosis-related genes, including BIM, FASL, TRAIL, and PUMA. Furthermore, FOXO controls autophagy through BNIP3, LC3 (MAP1LC3), and members of the ATG family, and also participates in the activation of stress response pathways, including the induction of HSP and SESN. Through these integrated and comprehensive networks, FOXO functions as a central hub that coordinates oxidative stress responses, cell cycle control, survival–death decision-making, and the maintenance of proteostasis. We note that the cells depicted in “Cell Differentiation” and “Apoptosis,” the autophagy diagram, the DNA in “DNA Repair” and “Stress Response” in Figure 1, and the SASP diagrams in Figure 2, Figure 3, and Figure 7 are similar to those in our previous publication [10].

In general, FOXO transcription factors regulate a broad range of target genes involved in antioxidant defense (superoxide dismutase 2 (SOD2), catalase (CAT), glutathione peroxidase 1 (GPX1), peroxiredoxin 3 (PRDX3)), cell cycle arrest (cyclin D, cyclin-dependent kinase 4 (CDK4), cyclin-dependent kinase inhibitor 1A (CDKN1A, p21), cyclin-dependent kinase inhibitor 1B (CDKN1B, p27)), DNA repair (growth arrest and DNA damage–inducible 45 (GADD45)), apoptosis (Bcl-2–interacting mediator of cell death (BCL2L11, BIM), Fas ligand (CD95L, FASL), TNF-related apoptosis-inducing ligand (TRAIL), p53 upregulated modulator of apoptosis (BBC3, PUMA)), autophagy (BCL2/adenovirus E1B 19 kDa protein–interacting protein 3 (BNIP3), microtubule-associated protein 1 light chain 3 (MAP1LC3, LC3), autophagy-related genes (ATG)), and stress responses (heat shock proteins (HSP), sestrins (SESN)) [14,15] (Figure 1). These findings have been demonstrated not only in in vitro systems such as mouse-derived fibroblasts and human colorectal cancer cell lines, but also across a wide range of pathological conditions, including human cancer tissues, neurodegenerative diseases, cardiovascular disorders, and metabolic diseases. FOXO transcription factors are evolutionarily conserved, and studies in animal models such as Hydra, nematodes (C. elegans), Drosophila, and mice suggest that they play a critical role in lifespan regulation [15]. Collectively, FOXO integrates cellular senescence, damage responses, and energy and oxidative stress management, thereby contributing to tissue homeostasis and maintenance of receptivity, while orchestrating the balance between cellular stress responses, survival, and death through multiple mechanisms.

The activity of FOXO1 is tightly regulated by multiple post-translational modifications. Phosphorylation by the phosphoinositide 3-kinase (PI3K)/protein kinase B (AKT) pathway promotes binding to 14-3-3 proteins, resulting in cytoplasmic translocation and transcriptional inactivation [16,17,18,19,20]. Additional regulatory layers include phosphorylation by NIMA (Never in Mitosis Gene A)-related kinase 2 (NEK2) [21], acetylation by CREB-binding protein/E1A-binding protein p300 (CBP/p300) [22], transcriptional upregulation of SIRT1 [23], deacetylation of FOXO1 by sirtuin 1 (SIRT1) [24], and activation via c-Jun N-terminal kinase (JNK) signaling [25]. These modifications collectively determine the balance among senescence, apoptosis, survival, and repair. In particular, feedback interactions among SIRT1, p53, and FOXO1 ensure coordinated regulation of cellular senescence and cytoprotection [23,26].

Among these multiple pathways, FOXO1 serves as a central transcription factor regulating differentiation, stress responses, apoptosis, and senescence during decidualization of the endometrium, and its expression fluctuates across the menstrual cycle [27,28,29,30,31]. During the secretory phase, FOXO1 expression is upregulated in a progesterone-dependent manner, promoting the differentiation of stromal cells into decidual cells. During the decidualization process, FOXO1 induces partial senescence, thereby creating an environment conducive to embryo implantation [30,32]. Concurrently, it induces apoptosis in a subset of cells. By regulating decidualization markers such as IGFBP1 and PRL, mediators of cell cycle arrest (p21, p27), and apoptotic pathways, FOXO1 maintains a delicate balance between differentiation and selective cell elimination [27,33,34]. In addition, the PI3K–AKT pathway [16], NEK2 [21], and SIRT1 [35] function as major upstream regulators controlling FOXO1 expression and nuclear activity in the endometrium.

Overall, in the normal endometrium, FOXO1 expression increases during the secretory phase and promotes decidualization and implantation by integratively regulating senescence, apoptosis, antioxidant defense, and differentiation. Partially senescent decidual cells contribute to a dynamic implantation environment, and dysregulation of this process may lead to tissue disruption and pathological progression [36].

#### 3.1.2. Aberrant FOXO1 Regulation and Pathogenesis in Endometriosis

In endometriosis, multiple lines of evidence suggest that the expression and activity of FOXO1, which normally serves as a central regulator of decidualization and cellular senescence, are suppressed at several regulatory levels. Impairment of FOXO1 function contributes to defective decidualization, reduced apoptosis, decreased tolerance to reactive oxygen species (ROS), aberrant local cellular senescence, and dysregulated senescence-associated secretory phenotype (SASP) activity, all of which collectively promote the survival and progression of ectopic endometrial cells [21,37,38].

Several molecular mechanisms are involved in the downregulation of FOXO1 expression. SRY-Box transcription factor 4 (SOX4) positively regulates FOXO1 by stabilizing the progesterone receptor (PGR). Accordingly, progesterone resistance and attenuation of PGR signaling lead to FOXO1 downregulation, resulting in abnormal expression of decidualization markers, including insulin-like growth factor binding protein 1 (IGFBP1) and prolactin (PRL), as well as dysregulation of cell cycle and apoptosis control [38]. In addition, downregulation of Notch signaling [39] and constitutive activation of the PI3K/AKT pathway [16] promote FOXO1 phosphorylation and nuclear export, thereby leading to its inactivation. Other molecules, such as calpain 7 (CAPN7) [40] and NEK2 [32], also facilitate cytoplasmic translocation of FOXO1, resulting in reduced expression of target genes required for cell cycle arrest, including p21 and p27. Furthermore, m^6^A modification of FOXO1 mRNA mediated by methyltransferase-like 3 (METTL3, N^6^-methyladenosine RNA methyltransferase) and dysregulation of progesterone receptor membrane component 1 (PGRMC1) signaling contribute to decreased FOXO1 expression and impaired senescence responses [32,37,41]. In endometriosis, increased SIRT1 expression further exacerbates disease progression by promoting progesterone resistance, senescence avoidance, and epithelial–mesenchymal transition (EMT) [42,43,44]. In contrast, suppression of SIRT1 by m^6^A (METTL3) promotes cellular senescence via FOXO and acts to restrain lesion progression [45]. In addition, in endometrial cells, FOXO1 has been shown to regulate apoptotic pathways and antioxidant genes, including SOD2, and post-translational modification (deacetylation) or downregulation of FOXO1 by SIRT1 is thought to contribute to apoptosis resistance and altered oxidative stress responses in endometriosis [27].

In summary, in the normal endometrium, FOXO1 coordinates decidualization and local senescence and maintains tissue homeostasis through appropriate regulation of apoptosis. In contrast, in endometriosis, downregulation and dysregulation of FOXO1 disrupt these processes, leading to defective decidualization, apoptosis resistance, aberrant senescence, and dysregulated SASP. The convergence of these cellular abnormalities promotes the persistence, excessive proliferation, and invasion of ectopic endometrial cells. Collectively, the FOXO1 pathway represents a critical molecular framework for understanding the pathophysiology of endometriosis and for identifying potential therapeutic targets.

### 3.2. Signaling Pathways Associated with FOXO

In this section, we synthesize the actions and functions of FOXO transcription factors based on a broad range of studies employing in vitro cultured cells, animal models, and human samples, rather than restricting our discussion to specific endometrial or endometriosis-derived cells. This approach aims to provide a conceptual framework for understanding the general mechanisms of FOXO-mediated intracellular signaling, oxidative stress responses, apoptosis and autophagy induction, and to contextualize these universal processes in relation to endometrium-specific biology. In the latter part of each subsection, we also summarize signaling pathways that have been validated in endometrial or endometriotic cells and tissues.

#### 3.2.1. The PI3K Pathway

In general, the PI3K/AKT/mTOR pathway constitutes a central signaling network that operates across a wide range of cell types, from normal cells to cancer cells and various cultured cell systems. In response to extracellular stimuli such as growth factors, cytokines, hormones, insulin-like growth factor (IGF), and receptor tyrosine kinases (RTKs), this pathway integratively regulates fundamental cellular functions, including proliferation, differentiation, survival, and metabolism [46]. PI3K converts the membrane lipid phosphatidylinositol 4,5-bisphosphate (PIP2) into phosphatidylinositol 3,4,5-trisphosphate (PIP3), which recruits and activates AKT at the plasma membrane. Activated AKT subsequently stimulates mTOR, which acts through two distinct complexes, mTORC1 (Mechanistic Target of Rapamycin Complex 1) and mTORC2 (Mechanistic Target of Rapamycin Complex 2), to promote protein synthesis, cell growth, metabolic activation, and inhibition of apoptosis [46]. In contrast, PTEN and the TSC1 (Tuberous Sclerosis Complex 1)/TSC2 (Tuberous Sclerosis Complex 2) complex function as negative regulators of this pathway [47]. Downstream of PI3K/AKT signaling, mTORC1 activates ribosomal protein S6 kinase (S6K) and eukaryotic translation initiation factor 4E-binding protein 1 (4EBP1) to enhance protein synthesis, while inhibiting glycogen synthase kinase 3 beta (GSK3β) to promote cell cycle progression, and suppressing Bcl-2–associated agonist of cell death (BAD) and caspase-9 to inhibit apoptosis [46]. In addition, mTORC2 promotes cell migration and invasion through cytoskeletal reorganization and enhances angiogenesis and lesion invasiveness via the induction of vascular endothelial growth factor (VEGF) and matrix metalloproteinases (MMPs) [48].

In endometriosis, the PI3K/AKT/mTOR pathway is frequently hyperactivated, and multiple mechanisms contribute to this aberrant activation [49,50,51] (Figure 2①). Estrogen enhances the expression and activity of RTKs and, through receptor-mediated actions, regulates both the PI3K/AKT pathway [52] and the FOXO signaling cascade [53]. As a consequence, cellular proliferation and survival are promoted, whereas apoptosis is suppressed and decidualization is impaired. Moreover, endometriotic lesions are enriched with local growth factors and cytokines, such as VEGF, IL-6, and TNF-α, which further activate the PI3K/AKT pathway as well as the NF-κB (nuclear factor kappa-light-chain-enhancer of activated B cells) pathway, thereby amplifying inflammatory signaling and driving disease progression [54]. Under hypoxic conditions, crosstalk between the PI3K/AKT pathway and hypoxia-inducible factor 1α (HIF-1α) regulates cellular adaptive processes, including survival, metabolism, angiogenesis, and epithelial–mesenchymal transition (EMT) [55,56], ultimately leading to activation of the AKT/mTOR pathway [57]. Collectively, the PI3K/AKT/mTOR cascade functions as a central regulator of proliferative and survival signaling in endometriosis and contributes to its pathophysiology through modulation of FOXO1, autophagy, and apoptosis.

**Figure 2:**
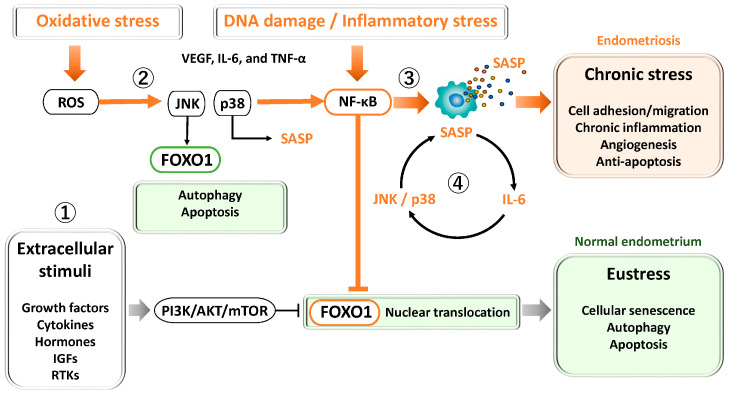
Key signaling pathways regulating FOXO1 and cellular stress responses in the endometrium and endometriosis. ① PI3K/AKT/mTOR pathway: Activated by growth factors, cytokines, and estrogen, this pathway promotes cell proliferation, survival, and metabolism while inhibiting apoptosis. In endometriosis, hyperactivation of PI3K/AKT/mTOR contributes to progesterone resistance, oxidative stress tolerance, and defective decidualization. ② JNK and p38 MAPK pathways: Transient activation under oxidative stress induces antioxidant defense and senescence during normal decidualization. In endometriosis, chronic activation leads to persistent inflammation, abnormal senescence, and apoptosis resistance through JNK/p38 and NF-κB–mediated anti-apoptotic signaling. ③ NF-κB pathway: Activated by oxidative stress, cytokines, and DNA damage, NF-κB induces inflammatory cytokines and SASP factors, supporting cell survival and angiogenesis. Chronic activation drives sustained inflammation, fibrosis, and lesion persistence. ④ STAT3 pathway: Activated by IL-6 and related cytokines via JAK signaling, STAT3 cooperates with NF-κB to amplify SASP production, forming a positive feedback loop that maintains chronic inflammation and senescence.

#### 3.2.2. JNK and p38

In general, in many cell types, mitogen-activated protein kinase (MAPK) pathways such as c-Jun N-terminal kinase (JNK), extracellular signal-regulated kinase (ERK), and p38 are activated in response to transient stimuli, including oxidative stress and DNA damage [58,59]. These pathways induce cell cycle arrest and certain senescence-like changes, while simultaneously promoting antioxidant responses and DNA repair through activation of FOXO transcription factors, thereby contributing to the maintenance of cellular homeostasis [25,60,61] (Figure 2②). JNK is a central kinase in cellular stress responses and induces apoptosis and autophagy through multiple pathways involving FOXO and p53; however, the effects of JNK are highly dependent on cell type and stimulus context [62]. In contrast, p38 MAPK is a key kinase driving senescence induction and regulates the upregulation of p21 and p16 as well as the formation of the senescence-associated secretory phenotype (SASP) through activation of multiple transcription factors, including p53, NF-κB, and CCAAT/enhancer-binding protein β (C/EBPβ) [63]. During the early phase of oxidative stress, p38 activates FOXO3a to promote the expression of antioxidant genes, thereby coordinating both the execution of cellular senescence and modulation of the surrounding microenvironment [64]. Through this coordinated FOXO activity, human endometrial stromal cells reportedly acquire resistance to oxidative stress–induced apoptosis during decidualization, thereby contributing to enhanced endometrial receptivity [27,28].

In contrast, in endometriotic tissues, multiple stimuli—including inflammation, oxidative stress, hormonal imbalance, and alterations in the local microenvironment—may form positive feedback loops that sustain chronic activation of JNK and p38 [51,65]. Persistent activation of these pathways accelerates the accumulation of senescent cells and the expression of senescence-associated phenotypes in endometriotic cells [66]. Stress responses that are normally reversible become pathologically dysregulated under the chronic inflammatory and estrogen-dominant environment characteristic of endometriosis. Furthermore, increased c-jun expression and reduced FOXO1 levels have been observed in endometriosis compared with eutopic endometrium [67], suggesting that dysregulation of the JNK/c-jun/FOXO1 axis may contribute to apoptosis suppression in endometriosis [68]. In addition, NF-κB, a key mediator of inflammatory responses, is activated in endometriosis; nuclear NF-κB induces anti-apoptotic genes (XIAP, Bcl-2, Bcl-xL), suppresses Bax/Bak, and inhibits cytochrome c release, thereby suppressing apoptosis. NF-κB also inhibits p53 activity and downregulates the expression of pro-apoptotic factors (PUMA, NOXA, Bax), potentially suppressing both extrinsic and intrinsic apoptotic pathways [68]. Moreover, in endometriotic cells and tissues, p38 promotes the establishment of a chronic inflammatory niche through SASP production [69,70,71]. As a consequence, cellular senescence in endometriosis shifts from a physiological “eustress” response to a pathological “chronic stress” response, leading to the irreversible accumulation of senescent cells and the maintenance of a persistent inflammatory microenvironment that increases the risk of implantation failure and infertility [69,70].

Thus, while JNK and p38 signaling act in an adaptive and transient manner to support reproductive function in the normal endometrium, these signaling pathways are chronically activated in endometriosis, driving a vicious cycle of cellular senescence and inflammation. The biological effects of JNK/p38 signaling appear to depend on both the intensity and duration of their activation. Accordingly, FOXO1, which is regulated by stress-response signaling pathways including JNK and p38, may exhibit distinct behaviors depending on the magnitude and nature of cellular stress. Although activation of p38 is a well-established finding in endometriosis research, to date there appear to be no studies that have explicitly examined the p38–FOXO axis in endometrial or endometriotic tissues.

#### 3.2.3. NF-κB and STAT3

General considerations, the transcription factor NF-κB is a central regulatory factor involved in diverse biological processes, including inflammatory responses, immune regulation, cell survival, differentiation, and tumorigenesis [72]. NF-κB responds to a wide range of stressors—such as DNA damage, oxidative stress, endoplasmic reticulum stress, hypoxia, metabolic dysregulation, and mechanical stimuli—and induces the transcription of inflammatory cytokines, thereby initiating the production of SASP. While this response promotes cellular survival and repair, chronic activation of NF-κB increases the risk of cellular senescence, tumorigenesis, and chronic inflammatory diseases [72].

In the normal endometrium, NF-κB–mediated regulation of SASP contributes to the maintenance of a receptive environment for embryo implantation [73] (Figure 2③). Age-associated upregulation of interleukin-17 receptor B (IL17RB) in endometrial epithelial cells activates the NF-κB pathway, resulting in sustained expression of SASP factors [73], thereby supporting the role of NF-κB in chronic inflammation and aging. NF-κB also contributes to the initiation, progression, and recurrence of endometriosis by regulating endometrial cell adhesion and migration (e.g., Cluster of Differentiation 44 (CD44), intercellular adhesion molecule 1 (ICAM-1), vascular cell adhesion molecule 1 (VCAM-1)), persistence of inflammatory responses (e.g., IL-1β, IL-6, IL-8, tumor necrosis factor alpha (TNF-α), interferon gamma (IFN-γ), regulated upon activation, normal T cell expressed and secreted (CCL5, RANTES)), angiogenesis (e.g., VEGF), and anti-apoptotic gene expression (e.g., Bcl-2, Bcl-xL, XIAP) [74].

By contrast, in endometriotic lesions, NF-κB signaling is constitutively activated and has been reported to contribute multifactorially to the pathogenesis of endometriosis, including chronic inflammation, invasion, angiogenesis, cell proliferation, and evasion of apoptosis, through the upregulation of inflammation-related genes [75,76]. In endometriotic cells, estrogen stimulation has been shown to activate the PI3K/Akt pathway via NF-κB activation, indicating the presence of functional crosstalk between these pathways [74]. Meanwhile, FOXO1 expression is significantly reduced in endometriosis patients compared with normal endometrium, which is thought to result from sustained hyperphosphorylation of AKT in endometriosis-derived stromal cells, thereby suppressing nuclear accumulation and transcriptional activity of FOXO1 [16,67]. Collectively, these findings suggest that constitutive activation of NF-κB signaling in endometriosis promotes an inflammatory milieu that facilitates activation of the PI3K–Akt pathway, thereby indirectly contributing to reduced nuclear activity and function of FOXO1. However, the molecular mechanisms by which NF-κB directly suppresses FOXO1 remain to be elucidated. Furthermore, the Janus Kinase (JAK)/Signal Transducer and Activator of Transcription 3 (STAT3) pathway is an environment-responsive signaling cascade activated by IL-6 family cytokines and growth factors. IL-6/JAK/STAT3 signaling has been shown to reprogram the inflammatory microenvironment through polarization of M1 macrophages [77] and to promote cell proliferation in endometriotic tissues [78]. In both endometriotic cells and cancer cells, NF-κB and STAT3 interact during stress responses and jointly regulate inflammation, immune responses, and cellular survival and proliferation [79,80,81,82]. IL-6 induced by NF-κB further activates the JAK/STAT3 pathway, thereby sustaining and amplifying the expression of IL-6 and other inflammatory genes (Figure 2④). In addition, inflammatory cytokines such as IL-1β and TNF-α have been reported to induce sustained activation of MAPK signaling pathways, including p38 and JNK, in endometriosis, which in turn promotes the production of IL-6 and IL-8. These signaling cascades are thought to form part of the pathogenic basis supporting chronic inflammation as well as the survival and proliferation of ectopic endometrial cells [83].

Moreover, the cGAS (cyclic GMP-AMP synthase)–STING (stimulator of interferon genes) signaling pathway is a defense mechanism that senses cytosolic DNA and induces innate immune responses. When chronically activated, this pathway may promote NF-κB–dependent inflammation, cellular senescence, autophagy, and tissue remodeling, thereby contributing to the pathogenesis of endometriosis [84]. Extracellular nuclear DNA and mitochondrial DNA (mtDNA) are recognized by the cGAS-STING pathway, leading to activation of TANK-binding kinase 1 (TBK1)–interferon regulatory factor 3 (IRF3) signaling and induction of type I interferons and NF-κB [84]. This signaling axis enhances autophagy in endometriotic lesions [84]. A positive feedback loop between NF-κB and p38 further amplifies the expression of inflammatory cytokines and chemokines (SASP), propagates senescence to neighboring cells via paracrine signaling, and promotes chronic inflammation and fibrosis. The cooperative loop between NF-κB and STAT3 represents one of the key determinants sustaining long-term SASP under conditions of chronic inflammation and aging.

Taken together, these findings indicate that NF-κB signaling is constitutively activated in endometriosis, concomitant with robust activation of the PI3K–AKT pathway. These pathways establish a chronic inflammatory and pro-survival signaling environment and may suppress the nuclear activity of FOXO1 through AKT pathway activation.

#### 3.2.4. P53

General considerations, under physiological conditions, oxidative stress and DNA damage activate the DNA damage response (DDR) through ATM (ataxia telangiectasia mutated) and ATR (ATM and Rad3-related), leading to transient cell cycle arrest via Chk1 (checkpoint kinase 1) and Chk2 (checkpoint kinase 2) kinases [85]. During this process, the p53–p21 pathway functions appropriately, allowing cells to undergo a reversible G1/S phase arrest [85,86]. In parallel, p16 is induced by oxidative stress, DNA damage, or excessive growth factor signaling and regulates the cell cycle by inhibiting cyclin-dependent kinase 4/6 (CDK4/6) activity [87] (Figure 3①).

In contrast, in endometriosis, both ectopic lesions and eutopic endometrium frequently exhibit γH2AX (phosphorylated H2A histone family member X)-positive nuclei and elevated expression of DDR-related genes such as growth arrest and DNA damage-inducible alpha (GADD45A), suggesting aberrant DNA damage responses and repair mechanisms [85,88]. Notably, reduced expression and activity of p53 have been consistently reported in endometriotic tissues [89,90,91,92]. Several mechanisms have been proposed to account for this reduction, including Mouse Double Minute 2 homolog (MDM2)-mediated degradation [89,93], promoter methylation or transcriptional repression mediated by microRNAs such as miR-30d-5p, miR-27a-3p, and miR-375 [94], as well as suppression under estrogen-dominant conditions [91] (Figure 3②). In various cancer cells, FOXO1 is known to promote the transcription of MDM2, which in turn ubiquitinates and targets p53 for degradation [95]. As a result, the FOXO1–MDM2–p53 axis attenuates the tumor-suppressive effects of p53, facilitating cell cycle progression and aberrant cell survival, thereby contributing to lesion maintenance. p53, p16, and MDM2 are key molecules involved in apoptosis and cell cycle regulation, and dysregulation of their expression has also been implicated in the initiation and progression of endometriosis [89]. In particular, increased MDM2 expression and functional impairment of p53 have been suggested to be associated with apoptosis evasion in ectopic endometrial cells [89]. Therefore, this regulatory mechanism may operate not only in cancer cells but also in endometriotic cells through similar processes.

Furthermore, oxidative stress and iron overload in endometriosis activate NF-κB, inducing inflammatory factors such as IL-6, IL-8, and MMPs, and may propagate senescent phenotypes to neighboring cells through “bystander senescence” [81] (Figure 3③). In addition, endometriosis is associated with increased p16 expression and a higher proportion of senescence-associated β-galactosidase (SA-β-gal)-positive cells, suggesting an enhancement of senescence markers [96]. However, other studies have reported dysregulated cell cycle control due to decreased p16 expression [89,97]. Because p16 functions both as a cell cycle inhibitor and as a marker that accumulates in senescent cells, its biological significance differs depending on expression levels: low expression reflects enhanced proliferation and loss of cell cycle control, whereas high expression indicates accumulation of senescent cells. Moreover, reduced Lamin B1 expression has also been observed in endometriosis [98], leading to nuclear envelope instability and chromatin dysregulation, thereby further reinforcing senescence-associated cell cycle arrest (Figure 3④). Once cellular senescence is initiated, activated Rb further suppresses Lamin B1 expression [98]. By contrast, in the normal endometrium, stable Lamin B1 expression preserves nuclear architecture and prevents excessive senescence and induction of inflammatory SASP. Thus, in endometriosis, prolonged stress and chronic inflammation may give rise to a pathological state in which p53 downregulation and aberrant fluctuations in p16 coexist within a distinctive senescent context.

In general, p53 determines cellular fate in a dose- and duration-dependent manner. Mild and transient activation induces temporary cell cycle arrest through p21 and DNA repair genes. Moderate and sustained activation promotes senescence, whereas strong activation or severe DNA damage triggers apoptosis via PUMA, Phorbol-12-Myristate-13-Acetate-induced protein 1 (NOXA), Bcl-2-associated X protein (BAX), and other effectors [99,100]. However, in endometriosis, downregulation of p53 limits apoptosis induction, thereby favoring senescence and inflammation and contributing to lesion persistence and progression.

Taken together, in endometriosis, suppression of the p53–DDR pathway, FOXO1-mediated activation of MDM2, dysregulation of p16, and SASP production by senescent cells occur concurrently, forming a complex senescence-associated network that promotes cell cycle dysregulation, chronic inflammation, and sustained lesion growth.

**Figure 3:**
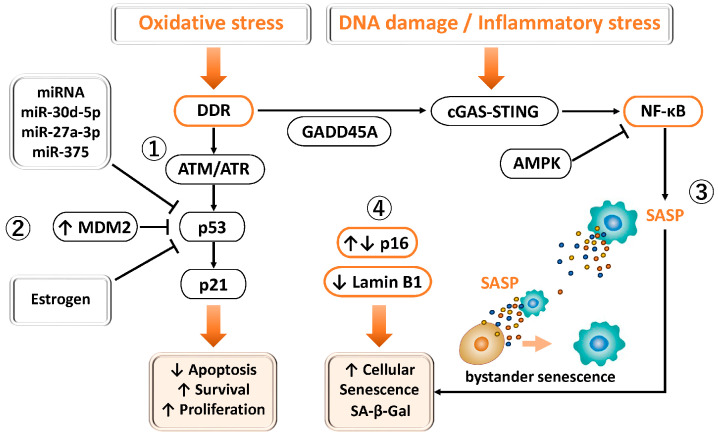
Involvement of p53 and FOXO1 in DNA damage response, senescence, and cell survival in endometriosis. ① Physiological DDR–p53 pathway: Under normal conditions, oxidative stress or DNA damage activates ATM/ATR–Chk1/Chk2 signaling, leading to p53-dependent induction of p21 and transient G1/S phase arrest. p16^INK4a also contributes to reversible cell cycle regulation under stress conditions. ② p53 suppression in endometriosis: In endometriotic tissues, p53 expression and activity are reduced through MDM2-mediated degradation, estrogen dominance, promoter methylation, and miRNA-mediated repression. MDM2 promotes p53 degradation and aberrant cell survival. This dysregulation attenuates DDR, apoptosis, and cell cycle arrest, thereby facilitating lesion persistence. ③ Senescence and inflammation: Altered p16 expression and loss of Lamin B1 are closely associated with cellular senescence and chronic inflammation, sustaining lesion growth. ④ Endometriosis exhibits dysregulated senescence and cell cycle control. Altered p16 expression reflects dual states, with low levels indicating enhanced proliferation and cell cycle deregulation, and high levels indicating accumulation of senescent (SA-β-gal–positive) cells. Reduced Lamin B1 further contributes to nuclear instability, chromatin dysregulation, and reinforcement of senescence-associated cell cycle arrest.

#### 3.2.5. SIRT1

In nematodes and mammalian cells, cancer cells, as well as various cultured cell models, SIRT1 is a nicotinamide adenine dinucleotide (NAD^+^)-dependent deacetylase that is known to regulate the transcriptional activity of FOXO transcription factors through deacetylation. In general, SIRT1-mediated deacetylation of FOXO1 promotes its nuclear localization and enhances the transcription of target genes, inducing the expression of antioxidant enzymes (e.g., SOD2, CAT), cell cycle regulators (p27, GADD45), and DNA repair–related genes [101,102]. Through these mechanisms, cells adapt to oxidative stress and DNA damage by activating cytoprotective pathways. Indeed, SIRT1-mediated deacetylation of FOXO has been reported to be essential for the induction of autophagy in cardiomyocytes in response to starvation stress [103].

**Figure 4:**
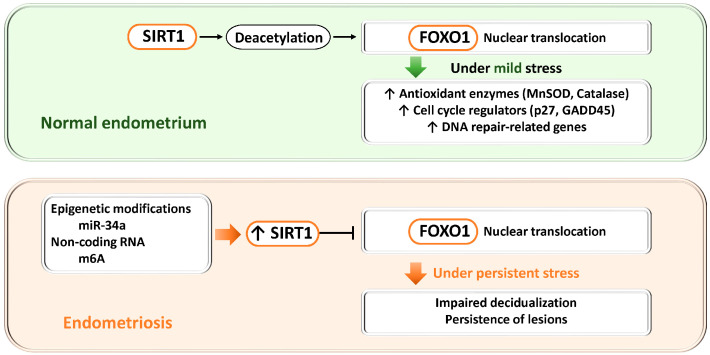
Dual roles of the SIRT1–FOXO1 axis in endometrial physiology and endometriosis. SIRT1, a NAD^+^-dependent deacetylase, regulates FOXO1 activity through deacetylation and thereby controls antioxidant defense, cell cycle regulation, and DNA repair. Upper panel (normal endometrium): When SIRT1 expression is low, FOXO1 transcription and nuclear activity are maintained, promoting decidualization, cell cycle arrest, apoptosis, and antioxidant responses required for endometrial receptivity and embryo implantation. Lower panel (endometriosis): Epigenetic alterations (miR-34a) and non-coding RNAs (m^6^A modification mediated by METTL3) increase SIRT1 expression. Excessive SIRT1 activity suppresses FOXO1 transcription and nuclear localization, impairs decidualization, and promotes lesion survival. Thus, physiological SIRT1–FOXO1 signaling supports cytoprotection and differentiation, whereas its dysregulation in endometriosis contributes to defective decidualization and disease persistence.

In the normal endometrium, SIRT1 expression is maintained at a low level, resulting in only weak repression of FOXO1 transcription, thereby preserving FOXO1 expression and nuclear activity [35] (Figure 4). Under these conditions, FOXO1 promotes decidualization and appropriately induces cell cycle arrest, apoptosis, and antioxidant responses, contributing to the formation of a receptive endometrium suitable for pregnancy.

In contrast, in endometriosis, epigenetic modification mediated by miR-34a has been suggested to regulate p53 via SIRT1 and to influence FOXO1 expression [104]. Moreover, non-coding RNAs and epigenetic modifications, such as METTL3-mediated m^6^A modification, have been reported to contextually regulate the activity of the SIRT1–FOXO1 axis [37,45]. In endometriotic lesions, SIRT1 expression is increased [42,44], which is considered part of a complex pathological state associated with epigenetic abnormalities. Excessive SIRT1 activity suppresses FOXO1 transcription and reduces its nuclear activity, leading to impaired decidualization and sustained lesion persistence.

Overall, while the SIRT1–FOXO1 axis in the normal endometrium functions to promote decidualization and cytoprotection, this axis exerts opposing effects in endometriosis and contributes to disease pathogenesis. This concept provides a unifying model linking dysregulation of SIRT1–FOXO1 signaling to endometrial dysfunction and lesion maintenance.

#### 3.2.6. mTOR

In general, SASP production is regulated at both the transcriptional and translational levels. At the transcriptional stage, transcription factors such as NF-κB [105] and C/EBPβ [106] induce the expression of major SASP-related genes. However, transcriptional activation alone is insufficient to drive robust protein production, making translational control indispensable. mTOR functions as a central regulator of this translational control. In particular, mTORC1 promotes translation initiation through phosphorylation of S6K and 4EBP1, thereby facilitating efficient translation of SASP-related mRNAs [107]. In addition, mTOR regulates the translation of IL1A [108] and mitogen-activated protein kinase-activated protein kinase 2 (MAPKAPK2, MK2) [109], indirectly modulating transcription factor activity and mRNA stability. Indeed, inhibition of IL1A translation by rapamycin has been shown to reduce NF-κB activity [108], supporting the notion that mTOR signaling is a critical regulator of the translational phase of SASP formation.

Furthermore, FOXO1 is a transcription factor that regulates autophagy in response to cellular energy status and stress signals, and its activity is closely linked to mTOR signaling [110]. FOXO1 induces the expression of SESN3, which inhibits mTORC1 via TSC2, thereby relieving autophagy suppression and promoting adaptive responses to metabolic stress [110]. FOXO1 also enhances the expression of Rictor, activating mTORC2 and forming a negative feedback loop in which AKT phosphorylation induces FOXO1 nuclear export [110]. For example, in macrophages within atherosclerotic lesions, mTORC2 has been reported to suppress FOXO1 expression itself, thereby influencing inflammatory responses and metabolic regulation [111]. In chondrocytes, FOXO1 promotes the transcription of autophagy-related genes such as ATG13 and Unc-51-like autophagy activating kinase 1 (ULK1), mediating adaptive responses to nutrient deprivation and oxidative stress [112].

**Figure 5:**
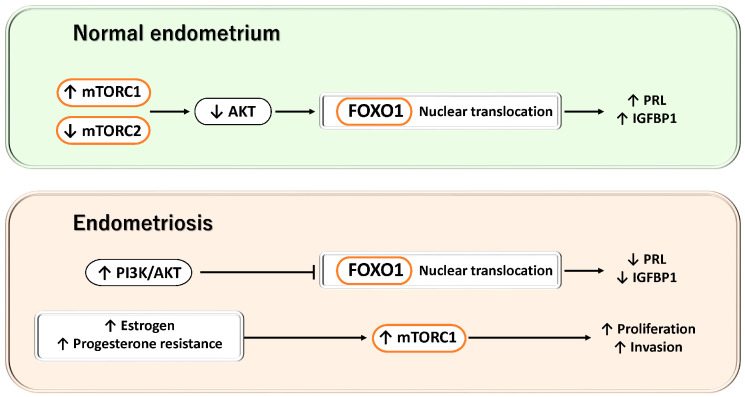
Dynamic regulation of the mTOR/AKT–FOXO1 axis during decidualization and its disruption in endometriosis. This figure illustrates the contrast between the decidualization process in normal endometrial stromal cells and the aberrant activation of PI3K/AKT/mTOR–FOXO1 signaling observed in endometriotic lesions. Upper panel: During normal decidualization, mTORC2 activity is reduced while mTORC1 activity is increased, leading to suppression of AKT phosphorylation. Consequently, FOXO1 phosphorylation is decreased, allowing FOXO1 to accumulate in the nucleus and enhance its transcriptional activity, thereby inducing the expression of decidualization-related genes such as PRL and IGFBP1. Lower panel: In endometriosis, estrogen stimulation, progesterone resistance, and constitutive activation of the PI3K/AKT pathway result in excessive activation of mTOR signaling, which promotes AKT-dependent phosphorylation and nuclear export of FOXO1. As a result, nuclear FOXO1 activity is reduced, the expression of IGFBP1 and PRL is suppressed, and defective decidualization ensues. Collectively, these findings indicate that the mTOR/AKT–FOXO1 axis plays a central role in the establishment of decidualization and in the pathophysiology of endometriosis.

In contrast, during the decidualization process of endometrial stromal cells, dynamic changes in mTOR signaling are observed. Specifically, mTORC2 activity decreases while mTORC1 activity increases, resulting in reduced AKT phosphorylation. Attenuation of AKT activity leads to decreased FOXO1 phosphorylation, allowing FOXO1 to accumulate in the nucleus and enhance its transcriptional activity, thereby inducing the expression of decidualization-related genes, including PRL and IGFBP1. This series of events represents evidence of the close crosstalk between mTOR signaling and FOXO1 activity during normal decidualization [113] (Figure 5).

Moreover, mTOR signaling forms extensive crosstalk with estrogen stimulation and the PI3K/AKT pathway, and enhanced mTOR responsiveness has been reported in benign endometrial disorders, including endometriosis [114]. Progesterone normally acts to suppress mTOR activity; however, in pathological conditions characterized by progesterone resistance, this inhibitory effect is disrupted, facilitating persistent proliferation of endometriotic cells. Hyperactivation of mTOR enhances the proliferative and invasive properties of endometrial cells, contributing to lesion formation and maintenance [114]. In addition, stromal cells derived from endometriotic lesions exhibit constitutive activation of the PI3K/AKT pathway, which promotes AKT-dependent phosphorylation and nuclear export of FOXO1, resulting in reduced nuclear FOXO1 levels and decreased expression of decidualization-related genes such as IGFBP1 and PRL [16]. These molecular alterations provide important evidence supporting a pathophysiological network in which constitutive activation of the PI3K/AKT/mTOR pathway suppresses FOXO1 activity, thereby leading to defective decidualization.

### 3.3. Regulatory Mechanisms of FOXO1 in the Pathophysiology of Endometriosis

**Figure 6:**
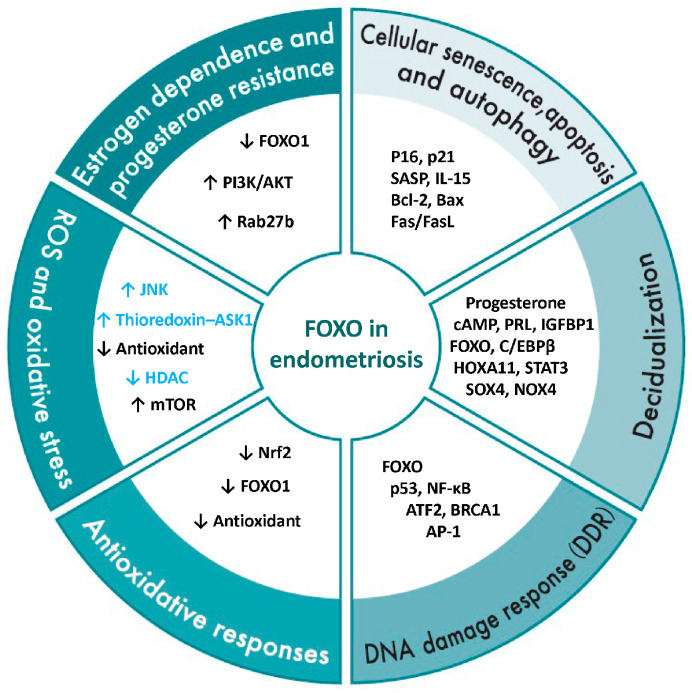
Overview of FOXO1 regulatory mechanisms in endometriosis. This figure illustrates the regulatory network of FOXO1 in the pathophysiology of endometriosis, organized into six major domains: estrogen dependency and progesterone resistance, reactive oxygen species (ROS) and oxidative stress, antioxidant responses, DNA damage response (DDR), decidualization, and cellular senescence, apoptosis, and autophagy. The genes, proteins, and signaling molecules listed within each domain represent FOXO1-related factors reported to contribute to these processes in endometriotic cells or tissues. Black text indicates FOXO-related factors that have been experimentally confirmed in normal endometrium or endometriotic tissues, while blue text denotes factors reported in other cell types or signaling contexts. Therefore, for factors shown in blue, their presence or function in endometriosis has not yet been experimentally validated. Collectively, these categories highlight the multifaceted role of FOXO1 as a central transcriptional regulator linking hormonal imbalance, oxidative stress, and abnormal cell fate decisions in endometriosis. Upward arrows indicate an increase or upregulation, whereas downward arrows indicate a decrease or downregulation.

This section describes the regulatory mechanisms of FOXO1 in the pathophysiology of endometriosis, categorized into estrogen dependence and progesterone resistance, ROS and oxidative stress, antioxidative responses, DNA damage response (DDR), decidualization, as well as cellular senescence, apoptosis, and autophagy (Figure 6).

#### 3.3.1. Estrogen Dependence and Progesterone Resistance

In the normal endometrium, decidualization is a differentiation process that functionally matures the endometrium in preparation for successful pregnancy and is tightly regulated by the coordinated actions of estrogen and progesterone [115]. During this process, FOXO1 is induced in a progesterone-dependent manner and functions as a central transcription factor that regulates the transcription of decidualization-related genes [33]. FOXO1 expression is increased in the secretory-phase endometrium and contributes to the progression of decidualization and the formation of the implantation window, while also being implicated in cyclic endometrial remodeling during the menstrual cycle and in protection against oxidative stress at the maternal–placental interface during pregnancy [67].

In contrast, reduced FOXO1 expression has been observed in endometriosis, and this reduction is suggested to contribute to impaired decidualization and decreased endometrial receptivity, thereby representing one of the potential causes of implantation failure and infertility [37,67]. These findings indicate that FOXO1 is an essential factor for normal decidualization and the maintenance of early pregnancy, while also highlighting its potential as a novel therapeutic target for endometriosis-associated infertility and pregnancy complications. In rats, an estrogen-dominant environment activates the PI3K/Akt pathway, leading to phosphorylation of FOXO1 and promotion of its nuclear export, thereby suppressing its transcriptional activity [116]. As a consequence, FOXO1-dependent functions, including antioxidant responses and the promotion of autophagy, are attenuated, resulting in the maintenance of cell survival and inflammatory responses. In the human endometrium, estrogen (17β-estradiol) has also been shown to activate the PI3K/Akt pathway through PI3K-dependent phosphorylation of Akt [117], and hyperactivation of this pathway is associated with reduced nuclear FOXO1 activity in human endometriotic cells [16]. Furthermore, in endometriosis, FOXO1 has been suggested to act as an upstream regulator of invasion and angiogenesis pathways mediated by the Ras-related protein Rab-27b (Rab27b), indicating that FOXO1 dysfunction may contribute to the invasive and progressive nature of endometriotic lesions [118]. Therefore, in endometriosis, persistent estrogen-dependent proliferation and inflammatory cytokine production, together with progesterone resistance–associated impairment of FOXO1 function, may cooperatively support the chronic survival, invasiveness, and angiogenesis of ectopic endometrial tissue.

#### 3.3.2. ROS and Oxidative Stress

In general, ROS activate thioredoxin–apoptosis signal-regulating kinase 1 (ASK1) [119] and the JNK pathway [25], thereby promoting the nuclear translocation of FOXO1. In addition, oxidation of PTEN and inactivation of AKT suppress the PI3K–AKT pathway [120], reduce cytoplasmic retention of FOXO1, and prolong its nuclear residence, leading to enhanced transcriptional activity. FOXO1 contains redox-sensitive cysteine residues, and reversible oxidation of these residues enhances its DNA-binding capacity and transcriptional activity [60]. Under conditions of moderate oxidative stress, FOXO1 induces antioxidant enzymes such as SOD2 and CAT, enabling cellular adaptation to oxidative environments [25,60]. Concurrently, inhibition of histone deacetylases (HDACs) and chromatin opening facilitate FOXO1 binding to the promoters of its target genes [121], thereby increasing the expression of decidualization markers such as PRL and IGFBP1. Thus, moderate levels of ROS act as important regulatory factors that promote decidualization through FOXO1-centered oxidative stress–responsive signaling pathways.

In endometrial stromal cells, decidualization stimuli such as cyclic adenosine monophosphate (cAMP) and progesterone enhance mitochondrial oxidative phosphorylation (OXPHOS), leading to increased ROS production from the electron transport chain. Mitochondria-derived ROS function not merely as byproducts but as signaling molecules that promote decidualization [122,123,124]. Indeed, decidualization of human endometrial stromal cells requires ROS derived from NADPH oxidases induced by cAMP signaling, and cAMP signaling has been shown to regulate the expression and function of the transcription factor FOXO1 and the progesterone receptor (particularly PR-A) in a ROS-dependent manner [122]. FOXO family transcription factors are involved in cellular antioxidant responses, regulation of apoptosis, and cell cycle control, and are closely associated with the process of endometrial decidualization [125]. In this context, ROS/oxidative stress and FOXO1 function are considered to be critically important for normal decidualization in the endometrium.

In contrast, in endometriosis, iron accumulation associated with retrograde menstruation [126] and macrophage activation [127,128] exacerbate local oxidative stress, while mitochondrial dysfunction sustains endogenous ROS production. These ROS activate the ERK and mTOR pathways [129], induce DNA damage and inflammatory cytokine secretion, and contribute to lesion proliferation and the maintenance of chronic inflammation [4]. Reduced FOXO1 expression in endometriosis is known to correlate with hyperactivation of the PI3K/AKT/mTOR pathway [16], and involvement of ferroptosis induced by iron-dependent lipid peroxidation has also been suggested [130].

Overall, in normal endometrial cells, mild ROS act as initiating signals for decidualization and fine-tune cellular adaptation to oxidative stress. FOXO functions as a “safety device” that utilizes ROS signaling while restraining damage caused by excessive ROS. In contrast, in endometriotic cells, FOXO1 is excluded from the nucleus, resulting in reduced transcriptional activity and diminished cellular adaptability to stress. When FOXO function is insufficient, ROS no longer act as physiological signaling molecules but instead function as pathological factors that drive persistent inflammation, fibrosis, promotion of cellular senescence, and ultimately defective decidualization. Thus, impairment of FOXO function is thought to be deeply involved in the exacerbation of excessive oxidative stress in endometriosis.

#### 3.3.3. Antioxidant Response

In general, cells respond to reactive oxygen species (ROS) and oxidative stress by activating antioxidant defense mechanisms, in which nuclear factor erythroid 2–related factor 2 (Nrf2) and FOXO1 function as major transcriptional regulators. Nrf2 is constitutively degraded through its interaction with Kelch-Like ECH-Associated Protein 1 (Keap1); however, modification of cysteine residues in Keap1 by reactive oxygen species or electrophiles disrupts this interaction, allowing Nrf2 to translocate into the nucleus and bind to antioxidant response elements (AREs). This induces the expression of a broad range of antioxidant genes, including heme oxygenase-1 (HO-1), SOD, CAT, glutathione (GSH), and NADPH quinone oxidoreductase 1 (NQO1) [131]. In addition, FOXO1, a central transcription factor within the antioxidant network, directly induces the expression of SOD2 [60,132], CAT [60,133], GPX1 [133], PRDX3 [134], SESN3 [110], and the DNA repair gene GADD45 [135]. Notably, SOD2 and PRDX3 are localized in mitochondria, where they remove superoxide and hydrogen peroxide, thereby protecting mitochondrial function. FOXO1 also coordinates multilayered defense mechanisms against oxidative damage through the induction of autophagy and DNA repair, and its activation in decidualized cells enhances protection against oxidative stress [10,27]. Depending on the intensity and nature of oxidative stress, FOXO1 dynamically shuttles between the nucleus and cytoplasm to fine-tune transcriptional activity and antioxidant responses. Moreover, FOXO1 transcriptional activity is precisely regulated by post-translational modifications mediated through multiple signaling pathways, including PI3K/AKT [20], JNK [25], AMPK, mTOR [110], and SIRT1 [24,136], all of which are therefore also involved in antioxidant responses.

In the human endometrium, progesterone signaling and FOXO1 transcriptional activity interact to regulate decidualization of endometrial stromal cells [125]. Upon decidualization, increased expression of antioxidant enzymes (such as SOD) confers resistance to oxidative damage [137]. In contrast, endometriotic tissues exhibit reduced expression of Nrf2 and its downstream effector glutamate–cysteine ligase (GCL), suggesting impairment of antioxidant responses [138]. In addition, in endometriotic lesions, sustained activation of the PI3K/AKT and SIRT1 pathways, together with chronic stimulation by inflammatory cytokines, promotes FOXO1 phosphorylation and nuclear export, resulting in reduced transcriptional activity [21,125]. Consequently, despite compensatory activation of repair mechanisms mediated by GADD45 [88], induction of antioxidant enzymes is insufficient, leading to mitochondrial ROS accumulation, increased oxidative DNA damage, and reduced genomic stability. Thus, in endometriosis, the coordinated antioxidant network mediated by Nrf2 and FOXO1 is disrupted, and ROS shift from acting as “physiological signals” to “pathological stressors.” This shift promotes chronic inflammation and cellular senescence while sustaining tumor-like proliferative and invasive capacities in endometriosis.

Overall, compared with normal endometrium, endometriosis is characterized by impaired FOXO function and breakdown of antioxidant responses, resulting in uncontrolled ROS accumulation that drives inflammation and fibrosis and compromises decidualization and endometrial receptivity. In this context, insufficient execution of the antioxidant defense role mediated by FOXO represents a key factor underlying the chronicity of endometriotic lesions, infertility, tissue fibrosis, and lesion persistence.

#### 3.3.4. DNA Damage Response (DDR)

In general, when DNA damage occurs, the p53–p21 pathway transiently arrests the cell cycle, thereby allowing DNA repair before proliferation resumes [85]. Multiple transcription factors are involved in the DNA damage response (DDR), including the FOXO family, p53, NF-κB, activating transcription factor 2 (ATF2), breast cancer susceptibility protein 1 (BRCA1), and activator protein-1 (AP-1) [139,140,141]. In particular, FOXO3a induces the expression of GADD45 [142] as well as DNA repair genes such as meiotic recombination 11 homolog (MRE11), RAD50 double-strand break repair protein (RAD50), RAD51 recombinase (RAD51), and BRCA1 [143]. FOXO transcription factors also activate the transcription of cyclin-dependent kinase inhibitors, antioxidant enzymes [132,133], and autophagy-related factors [144,145], thereby protecting cells from oxidative stress. In addition, AKT inhibits the nuclear translocation of FOXO while mediating the nuclear localization of human telomerase reverse transcriptase (hTERT), thereby promoting the survival and proliferation of damaged cells [146].

In normal endometrial stromal cells, induction of FOXO1 enhances oxidative stress defense, whereas suppression of FOXO3a prevents excessive apoptosis, thereby maintaining cellular homeostasis [27]. In contrast, endometriotic lesions are characterized by persistent chronic inflammation and excessive ROS, leading to increased damage to nuclear DNA and mitochondrial DNA within endometrial tissues [147]. At the same time, endometriotic cells show reduced expression and/or activity of DNA repair mechanisms, including the ATM/ATR and BRCA/RAD pathways [148]. The accumulation of these defects results in a state in which DNA damage is inefficiently repaired, potentially establishing a vicious cycle of chronic inflammation and lesion progression [85]. Moreover, in endometriosis, sustained ROS elevation activates the PI3K/AKT pathway, promotes nuclear export of FOXO1, and suppresses the transcription of antioxidant and DNA repair genes [21,60,68]. Nuclear exclusion of FOXO factors permits the survival of damaged cells and facilitates chronic proliferation and persistent inflammation of ectopic endometrial tissue. In addition, activating factors such as cytosolic DNA or mtDNA–mediated activation of the cGAS–STING pathway [84] establish positive feedback loops involving autophagy, further stabilizing the chronic inflammatory microenvironment. Such disruption of DDR impairs decidualization capacity and provides a molecular basis for implantation failure and infertility, positioning DDR dysfunction as a critical mechanism underlying reproductive impairment in endometriosis.

Overall, whereas antioxidant and repair responses centered on FOXO1 maintain homeostasis in the normal endometrium, in endometriosis, ROS-mediated suppression of FOXO activity and impaired repair responses synergistically promote the survival of damaged cells and chronic inflammation. Under pathological conditions such as endometriosis, increased PI3K–AKT activity and the effects of chronic inflammation suppress FOXO- and p53-dependent apoptosis, rendering damaged cells resistant to elimination and thereby contributing to disease pathogenesis. Collectively, these findings suggest that therapeutic strategies aimed at reactivating the FOXO pathway may hold promise for the treatment of endometriosis.

#### 3.3.5. Decidualization

Decidualization of the endometrium is an essential differentiation process required for embryo implantation and the maintenance of pregnancy and is regulated by a multilayered network comprising hormones, transcription factors, cell cycle regulators, and metabolic signaling pathways [33]. The central master transcription factor in this process is FOXO1, which is activated in response to progesterone and cAMP stimulation and directly induces the expression of decidual markers such as PRL and IGFBP1 [33,149]. In the normal endometrium, PGR signaling and the cAMP/protein kinase A (PKA) pathway promote FOXO1 transcription and nuclear localization [28], and FOXO1 cooperates with C/EBPβ [150], homeobox A11 (HOXA11) [151], and STAT3 [152] to induce the expression of differentiation-associated genes. SOX4 promotes decidualization by maintaining PGR stability and regulating FOXO1 expression [38]. In addition, moderate levels of NADPH oxidase 4 (NOX4)–derived ROS act as signaling molecules that modify FOXO1 and C/EBPβ [122]. Meanwhile, the AMPK and mTOR pathways regulate FOXO1 stability, thereby enabling differentiation in accordance with the cellular metabolic state [113]. These coordinated actions result in cell cycle arrest, induction of PRL and IGFBP1 expression, and establishment of an endometrial receptive environment suitable for pregnancy [33].

In contrast, this regulatory machinery is disrupted at multiple levels in endometriosis. First, comparison of eutopic endometrium from patients with endometriosis and control subjects revealed a reduced proportion of cells exhibiting strong or moderate positivity for PGRMC1 protein during the proliferative phase in patients with endometriosis [153]. In human endometrial cells, reduced PGRMC1 expression increases FOXO1 levels and promotes cellular senescence during decidualization [32,154]. Conversely, conditional knockout mouse models of FOXO1 exhibit loss of cell polarity and abnormal apoptosis, leading to implantation failure and infertility via dysregulated catenin beta 1 (CTNNB1) signaling [155]. Moreover, abnormalities in upstream regulators of FOXO1 have also been reported, including sustained activation of AKT/mTOR signaling [16], dysregulated cell cycle control mediated by NEK2 [21], impaired regulation of cyclooxygenase-2 (COX2) and cAMP-responsive genes via PGRMC1 [32,154], FOXO1 exclusion through AKT cleavage mediated by CAPN7 [40], aberrant m^6^A modification by METTL3 [37], dysfunction of the Notch pathway [39], modulation of bone morphogenetic protein (BMP) signaling [156], and reduced expression of PRMT5 [157]. Collectively, these alterations suppress FOXO1-dependent differentiation signaling, namely decidualization, while enhancing survival signaling, thereby promoting the pathological persistence of ectopic endometrial tissue. In general, in the normal endometrium, decidualization is promoted by progesterone/cAMP-mediated nuclear retention and transcriptional activation of FOXO1. In contrast, in endometriosis, FOXO1 function is suppressed by the combined effects of reduced progesterone production, overexpression of estrogen receptor beta (Erβ), and abnormalities in upstream regulatory factors, ultimately leading to decidualization failure. This represents a major molecular basis for implantation failure and infertility.

Taken together, FOXO transcription factors are indispensable for decidualization of human endometrial stromal cells, inducing the expression of key markers such as PRL and IGFBP1 and maintaining endometrial homeostasis. However, in endometriotic cells, excessive activation of pathways such as PI3K–AKT promotes nuclear export of FOXO1 and reduces its transcriptional activity. Consequently, decidualization is insufficient, endometrial receptivity is compromised, and the risk of implantation failure, infertility, and recurrent miscarriage is increased. In addition, impairment of FOXO function is thought to contribute to the progression of endometriosis pathology, including inflammation, fibrosis, and estrogen-dependent aberrant proliferation.

#### 3.3.6. Cellular Senescence, Apoptosis, and Autophagy

In general, FOXO functions as a central regulator of autophagy and maintains cellular homeostasis through both transcriptional and non-transcriptional mechanisms in coordination with the mTOR–AMPK axis. For example, in muscle cells and other cell types, FOXO1 coordinately regulates the transcription of autophagy-related genes such as the ATG family, Beclin-1, and LC3, thereby facilitating the removal of damaged mitochondria and maintaining ROS homeostasis to preserve cellular function [145,158,159].

In the normal human endometrium, decidualization induces a subset of stromal cells to activate FOXO1-dependent transcription of cell cycle inhibitors such as p16 and p21, antioxidant enzymes, and SASP components, thereby regulating immune cell recruitment, angiogenesis, and extracellular matrix remodeling [30]. Moreover, the coordinated regulation of senescence, apoptosis, and autophagy in a FOXO1-dependent manner is essential for decidualization and preparation for pregnancy, and IL-15–activated uterine natural killer (uNK) cells promote the rapid clearance of senescent cells to maintain tissue homeostasis [30]. Indeed, in porcine endometrial epithelial cells, it has been shown that under mild oxidative stress, FOXO1 promotes cytoplasmic autophagy through interaction with ATG7, whereas under severe oxidative stress, nuclear translocation of FOXO1 activates caspase pathways and induces apoptosis. In this manner, the balance among senescence, autophagy, and apoptosis is dynamically maintained in accordance with cellular status [160].

In contrast, in endometriosis, persistent oxidative stress and mitochondrial dysfunction lead to excessive ROS production, constitutive activation of signaling pathways such as PI3K/Akt, MAPK, NF-κB, and mTOR [49], insufficient antioxidant responses, dysregulated senescence control, and abnormal autophagy. These alterations promote chronic SASP secretion, reduced autophagy, enhanced cell survival, and sustained proliferation and inflammation of ectopic endometrial tissue [161,162,163,164,165]. Furthermore, overexpression of Bcl-2 and Bcl-xL [166], suppression of Bax/Bcl-2 homologous antagonist/killer (Bak) and caspase pathways [167], attenuation of Fas/FasL signaling [168], ERβ-dependent anti-apoptotic signaling [169], and reduced FOXO1 activity collectively facilitate the survival of ectopic cells and may link dysregulation of senescence, apoptosis, and autophagy to lesion persistence and chronic inflammation. Multiple cell types, including endometrial stromal cells [11,170], epithelial cells [161,171], and macrophage populations [172,173], contribute to disease progression through SASP, epithelial–mesenchymal transition (EMT), and fibrosis. Dysregulation of SIRT1-FOXO1 signaling has also been observed in rat models of endometriosis [136]. FOXO1 functions as a critical molecular switch regulating the balance between autophagy and apoptosis [160], and elucidating the regulatory mechanisms of FOXO1 in human endometriotic cells remains an important research objective. Overall, FOXO1 plays a central role in integrating senescence, apoptosis, and autophagy in the endometrium, and its functional impairment in endometriosis directly contributes to chronic inflammation, survival of ectopic tissue, and reduced fertility, highlighting its potential as a therapeutic target.

In summary, reduced FOXO activity in endometriosis leads to multiple abnormalities, including ROS accumulation, disruption of redox homeostasis, DDR dysfunction, impaired regulation of cellular senescence, attenuated apoptotic responses, and imbalanced autophagy, ultimately resulting in defective decidualization. These changes promote the persistence and fibrosis of estrogen-dependent inflammatory lesions, directly impair endometrial receptivity, and contribute to implantation failure and infertility. In other words, in endometriosis, the multifaceted functions of FOXO—stress adaptation, cellular quality control, and promotion of decidualization—are not fully realized, and this functional impairment is a major factor exacerbating the disease.

## 4. Discussion

FOXO proteins are transcription factors that integrate cellular stress responses and play central roles in the regulation of decidualization, cellular senescence, autophagy, and apoptosis in the endometrium [33,160]. In endometriosis, reduced FOXO1 activity promotes the proliferation of ectopic endometrial cells while suppressing apoptosis and damage responses, thereby contributing to lesion expansion and chronic inflammation. FOXO1 maintains the balance between cell survival and cell death and contributes to the pathogenesis of endometriosis in a multifaceted manner.

**Figure 7:**
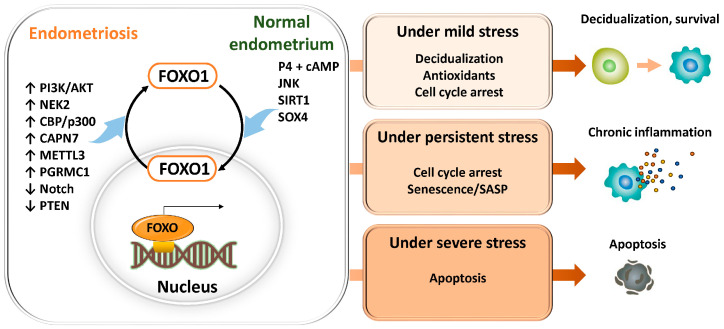
FOXO1 regulation of decidualization, senescence, autophagy, and apoptosis in the endometrium and endometriosis. FOXO1 integrates cellular stress signals to control cell fate in the endometrium. FOXO1 activity is regulated by post-translational modifications, mRNA methylation (METTL3/YTHDF2), cofactors (Notch, SOX4), and upstream pathways (SIRT1, AMPK, PI3K/AKT, estrogen). In endometriosis, dysregulation of these mechanisms impairs decidualization, promotes senescence and SASP, and suppresses apoptosis, facilitating lesion persistence. Under mild stress or progesterone, it transiently enters the nucleus, inducing decidualization genes (PRL, IGFBP1), antioxidants (MnSOD, catalase), and cell cycle inhibitors (p21, p27), while promoting autophagy via ATG7, supporting differentiation and survival. Under persistent oxidative stress or DNA damage, FOXO1 drives senescence by upregulating p21, p16, and SASP factors (IL-6, IL-8, MMPs), enhancing chronic inflammation through NF-κB and p53 crosstalk. Under severe stress conditions, FOXO1 may influence apoptosis via Bim and FasL, depending on the balance between JNK/p38 and PI3K-AKT activation.

The nuclear dynamics and transcriptional activity of FOXO1 vary depending on the type, intensity, and duration of stimuli, the metabolic and hormonal environment of the cell, and crosstalk with other transcription factors, ultimately determining distinct cell fates (Figure 7). Under mild stress or hormonal stimulation such as progesterone, FOXO1 transiently translocates to the nucleus and induces the expression of decidualization-related genes (PRL, IGFBP1), antioxidant enzymes (SOD2, CAT), and cell cycle inhibitors (p21, p27), thereby promoting differentiation and cell survival [27,155,174]. In the cytoplasm, FOXO1 interacts with ATG7 to activate autophagy and enhance cellular defense against oxidative stress [160].

Conversely, under conditions of persistent oxidative stress or DNA damage, FOXO1 remains in the nucleus for prolonged periods and induces cell cycle arrest factors (p21, p16) and SASP components (IL-6, IL-8, MMP), leading to the establishment of a senescent phenotype and promoting chronic inflammation and tissue remodeling through crosstalk with NF-κB and p53 [30,161,170]. Furthermore, studies using cancer cells and mouse granulosa cells have demonstrated that under severe oxidative stress or phosphorylation mediated by the JNK/p38 MAPK pathway, apoptosis-related genes such as BIM and FasL are activated, resulting in caspase-dependent cell death [175,176,177,178]. In normal human endometrial stromal cells, FOXO3a has also been reported to be induced by oxidative stress and to promote apoptosis [27]. However, in the endometrium and endometriosis, despite persistent oxidative stress or MAPK activation, counteracting survival signals such as PI3K-AKT are often strongly activated, leading in many cases to suppression of BIM/FasL and the development of apoptosis resistance [66]. Thus, in endometriosis, FOXO1 is thought overall to regulate cell fate primarily by branching toward “decidualization and cell survival under mild stress” and “cellular senescence and SASP formation under persistent stress,” while inducing “apoptosis under severe stress” only in limited and exceptional circumstances.

The nuclear retention and transcriptional activity of FOXO1 are regulated by post-translational modifications (acetylation, deacetylation, phosphorylation), interactions with cofactors and chromatin regulators, mRNA modifications (m^6^A mediated by METTL3), and upstream transcription factors such as Notch and SOX4 [21,37,102,179,180,181]. SIRT1 and AMPK activities promote nuclear retention of FOXO1 and induction of antioxidant genes, whereas the PI3K/AKT pathway and estrogen-dominant environments facilitate FOXO1 phosphorylation, nuclear export, and suppression of its transcriptional activity [27,116,180,182]. Although many of these detailed mechanistic insights are primarily derived from studies using cancer cells, similar molecular signaling abnormalities and functional trends have also been reported in endometriosis [10,21,27,37,66,96,102,116,161]. As a result, FOXO1-dependent regulation is impaired in endometriosis, leading to reduced decidualization, accumulation of senescent cells, chronic inflammation, and apoptosis resistance, which collectively promote the survival of ectopic tissue and disease progression [10,66,96,161].

This review has several important limitations. First, the functional redundancy and compensatory interactions between FOXO1 and other FOXO family members have not been sufficiently examined, making it difficult to clearly distinguish whether alterations in a single factor are causal or represent part of broader pathway crosstalk. In addition, studies using clinical specimens are often limited by small sample sizes, and important background factors such as lesion location, hormonal environment, and inflammatory status are not always adequately controlled, necessitating cautious interpretation and generalization of the results. Moreover, given that endometriosis consists of heterogeneous cell populations, existing clinical studies based mainly on bulk analyses may fail to capture cell type–specific regulation of FOXO1. Therefore, improving the quality and quantity of clinical specimens, performing functional analyses at the level of individual cell populations, and conducting in vivo validation remain important future challenges for achieving a more precise understanding of FOXO1 regulation and its pathophysiological significance.

In summary, FOXO1 responses in the normal endometrium support cyclical regeneration and maintenance of pregnancy, whereas disruption of the FOXO1 regulatory network plays a central role in the pathogenesis of endometriosis. FOXO1 represents a promising therapeutic target through interventions such as modulation of mRNA modification, inhibition of the AKT pathway, and reprogramming of transcription factor networks. Quantitative evaluation of its nuclear retention and transcriptional activity will be essential for elucidating disease mechanisms and developing novel therapeutic strategies.

## 5. Future Perspectives

As highlighted in this review, FOXO1 occupies a central position in the regulation of cell fate in endometriosis, driving diverse branching responses—including decidualization, senescence, and apoptosis—depending on its nuclear retention time and activation pathways. However, several critical questions remain for future research.

First, quantitative visualization of FOXO1 nuclear retention dynamics is needed. While previous studies have shown condition-dependent changes in localization, it remains unclear how the duration of nuclear residency determines the decision between differentiation, senescence, or apoptosis. The implementation of live-cell imaging and single-cell analyses is expected to enable precise quantification of these dynamics.

Second, a comprehensive understanding of post-translational and RNA modification-mediated regulation is required. Although acetylation, phosphorylation, and m^6^A modifications have been individually reported, these modifications act in combination to govern FOXO1 dynamics and transcriptional activity. Proteomics- and epitranscriptomics-based approaches that integrate these modifications are essential for clarifying disease-dependent regulatory abnormalities.

Third, elucidation of crosstalk with upstream signaling pathways and transcription factor networks is crucial. While pathways such as AKT, Notch, and SOX4 have been implicated in FOXO1 regulation, how these signals integrate with the hormonal milieu and inflammatory signals remains unknown. Interactions with estrogen receptors, p53, and NF-κB may be particularly relevant, linking inflammation with tumorigenic risk and holding potential clinical significance.

Moreover, the therapeutic applicability of targeting FOXO1 represents a major future challenge. Beyond direct modulation of FOXO1 activity, indirect interventions via mRNA modifications, upstream signaling regulation, or interactions with chromatin-modifying factors could serve as novel treatment strategies. Such approaches may also lay the foundation for precision medicine, stratifying patients by endometriosis subtype, age, or hormonal responsiveness.

In summary, further refinement of a FOXO1 branching response model promises not only to deepen mechanistic understanding of endometriosis pathophysiology but also to have broader implications for regenerative medicine, infertility treatments, and cancer prevention. Future studies focusing on spatiotemporal analyses of molecular dynamics, integrated networks of modifications, and validation of therapeutic targeting will be essential forward-looking priorities.

## Data Availability

No new data were created or analyzed in this study. Data sharing is not applicable to this article.

## Abbreviations

14-3-3	14-3-3 Proteins
4EBP1	Eukaryotic Translation Initiation Factor 4E-Binding Protein 1
AKT	Protein Kinase B (PKB)
AMPK	AMP-Activated Protein Kinase
AP-1	Activator Protein 1
ASL	Fas Ligand (CD95L)
ATF2	Activating Transcription Factor 2
ATGs	Autophagy-related Genes
AREs	Antioxidant response elements
ASK1	Apoptosis Signal-Regulating Kinase 1
ATM	Ataxia-telangiectasia mutated
ATR	ATM and Rad3-related
BAD	Bcl-2-Associated Death Promoter
Bak	Bcl-2 Homologous Antagonist/Killer
BAX	Bcl-2-Associated X Protein
Bcl-2	B-cell lymphoma 2
Bcl-xL	B-cell lymphoma-extra large
BH3	Bcl-2 Homology 3
BIM	Bcl-2 Interacting Mediator of Cell Death (BCL2L11)
BMP	Bone Morphogenetic Protein
BNIP3	BCL2/adenovirus E1B 19 kDa Protein-Interacting Protein 3
BRCA1	Breast Cancer Type 1 Susceptibility Protein
cAMP	Cyclic Adenosine Monophosphate
CAPN7	Calpain 7
CAT	Catalase
CBP/p300	CREB-Binding Protein/E1A Binding Protein p300
CD44	Cluster of Differentiation 44
CDK4	Cyclin-Dependent Kinase 4
C/EBPβ	CCAAT/Enhancer-Binding Protein Beta
Chk1	Checkpoint Kinase 1
Chk2	Checkpoint Kinase 2
COX2	Cyclooxygenase-2
CTNNB1	Catenin Beta 1
DDR	DNA damage response
EMT	epithelial–mesenchymal transition
ERβ	Estrogen Receptor Beta
ERK	Extracellular Signal-Regulated Kinase
FHL2	Four and a half LIM domain protein 2
FOXO	Forkhead box O
GADD45	Growth Arrest and DNA Damage-inducible 45
GADD45A	Growth Arrest and DNA Damage-Inducible Alpha
GCL	Glutamate–Cysteine Ligase
GSH	Glutathione
GPX1	Glutathione Peroxidase 1
HDAC	Histone deacetylase
HIF-1α	Hypoxia-Inducible Factor 1 Alpha
HO-1	Heme oxygenase-1
HOXA11	Homeobox A11
HSPs	Heat Shock Proteins
hTERT	Human Telomerase Reverse Transcriptase
ICAM-1	Intercellular Adhesion Molecule 1
IFN-γ	Interferon Gamma
IGFs	Insulin-like growth factors
IGFBP1	Insulin-Like Growth Factor Binding Protein 1
IL17RB	Interleukin 17 Receptor B
IRF3	Interferon Regulatory Factor 3
JAK	Janus Kinase
JNK	c-Jun N-terminal Kinase
Keap1	Kelch-Like ECH-Associated Protein 1
LC3	Microtubule-associated protein 1 light chain 3 (MAP1LC3)
MAPKAPK2	Mitogen-Activated Protein Kinase-Activated Protein Kinase 2
METTL3	Methyltransferase-Like 3 (N^6^-methyladenosine RNA methyltransferase)
MDM2	Mouse Double Minute 2 homolog
miRNAs	MicroRNAs
MMPs	Matrix Metalloproteinases
MRE11	Meiotic Recombination 11 Homolog
mtDNA	mitochondrial DNA
mTOR	Mechanistic target of rapamycin
mTORC1	Mechanistic Target of Rapamycin Complex 1
mTORC2	Mechanistic Target of Rapamycin Complex 2
NAD	Nicotinamide Adenine Dinucleotide
NEK2	NIMA (Never in Mitosis Gene A)-Related Kinase 2
NF-κB	Nuclear Factor kappa-light-chain-enhancer of activated B cells
NOXA	Phorbol-12-Myristate-13-Acetate-Induced Protein 1 (PMAIP1)
NOX4	NADPH Oxidase 4
NQO1	NADPH quinone oxidoreductase 1
Nrf2	Nuclear Factor Erythroid 2–Related Factor 2
OXPHOS	Mitochondrial oxidative phosphorylation
p21	Cyclin-Dependent Kinase Inhibitor 1A (CDKN1A)
p27	Cyclin-Dependent Kinase Inhibitor 1B (CDKN1B)
PGRMC1	Progesterone Receptor Membrane Component 1
PI3K	Phosphoinositide 3-Kinase
PIP2	Phosphatidylinositol 4,5-bisphosphate
PIP3	Phosphatidylinositol 3,4,5-trisphosphate
PKA	Protein Kinase A
γH2AX	Phosphorylated H2A Histone Family Member X
PRDX3	Peroxiredoxin 3
PRMT5	Protein Arginine Methyltransferase 5
PRL	Prolactin
PTEN	Phosphatase and Tensin Homolog
PUMA	p53 Upregulated Modulator of Apoptosis (BBC3)
Rab27b	Ras-Related Protein Rab-27b
RAD50	RAD50 Double Strand Break Repair Protein
RAD51	RAD51 Recombinase
RANTES	Regulated on Activation, Normal T Cell Expressed and Secreted (CCL5)
RTKs	Receptor tyrosine kinases
S6K	Ribosomal Protein S6 Kinase
SA-β-gal	Senescence-Associated Beta-Galactosidase
SASP	Senescence-Associated Secretory Phenotype
SESN	Sestrins
SIRT1	Sirtuin 1 (NAD^+^-dependent deacetylase)
SOD2	Superoxide Dismutase 2 (MnSOD)
SOX4	SRY-Box Transcription Factor 4
STAT3	Signal Transducer and Activator of Transcription 3
STING	Stimulator of Interferon Genes
TBK1	TANK-Binding Kinase 1
TNF-α	Tumor Necrosis Factor Alpha
TRAIL	TNF-Related Apoptosis-Inducing Ligand
TSC1	Tuberous Sclerosis Complex 1
TSC2	Tuberous Sclerosis Complex 2
ULK1	Unc-51-Like Autophagy Activating Kinase 1
uNK	Uterine Natural Killer (Cells)
VCAM-1	Vascular Cell Adhesion Molecule 1
VEGF	Vascular Endothelial Growth Factor
WIP1	Wild-Type p53-Induced Phosphatase 1
XIAP	X-linked inhibitor of apoptosis protein
